# Trends in Apomixis Research: The 10 Most Cited Research Articles Published in the Pregenomic and Genomic Eras

**DOI:** 10.3389/fpls.2022.878074

**Published:** 2022-05-03

**Authors:** Fabio Palumbo, Samela Draga, Alessandro Vannozzi, Margherita Lucchin, Gianni Barcaccia

**Affiliations:** Laboratory of Plant Genetics and Breeding, Department of Agronomy, Food, Natural Resources, Animals and Environment (DAFNAE), University of Padua, Padua, Italy

**Keywords:** asexual reproduction, MMC, meiosis, model species, bibliometric analysis

## Abstract

Apomixis, or asexual reproduction by seed, represents an easy shortcut for life cycle renewal based on maternal embryo production without ploidy reduction (meiosis) and ploidy restitution (syngamy). Although the first studies officially published on this topic in scientific journals date back to the early 1930s, the identification and introduction of genes involved in asexual reproduction in species of agronomic interest still represent a major challenge. Through a bibliometric analysis of the research programs implemented in apomixis over the last 40 years, the present study was aimed to discuss not only the main findings achieved but also the investigational methods and model species used. We split the critical survey of the most cited original articles into pregenomic and genomic eras to identify potential trends and depict scenarios that have emerged in the scientific community working on apomixis, as well as to determine any correlation between the exponential increase in acquired basic knowledge and the development of advanced analytical technologies. This review found a substantial stagnation in the use of the same model species, with few exceptions, for at least 40 years. In contrast, the development of new molecular techniques, genomic platforms, and repositories has directly affected the approaches used in research, which has been directed toward an increasingly focused study of the genetic and epigenetic determinants of apomixis.

## Introduction

Apomixis is highly desirable in agriculture as a reproductive strategy for cloning plants by seeds (Nogler, 1984). Because embryos derive from the parthenogenic development of apomeiotic egg cells, apomixis results in offspring that are exact genetic replicas of the parent. Introgression of apomixis from wild relatives to crop species and transformation of sexual genotypes into apomictically reproducing ones are long-held goals of plant breeding. It is generally accepted that the introduction of apomixis into agronomically important crops has revolutionary implications for agriculture. In recent years, many scientists have speculated on the isolation of genes controlling key steps of the apomictic pathway. Moreover, several papers have postulated the production of engineered plants exhibiting apomictic-like phenotypes. However, in model species only some features of apomixis have been genetically engineered and none of the major crop plants has been edited or bred for apomixis (for review see Albertini et al. (2010), Pupilli and Barcaccia (2012), Vijverberg et al. (2019), Hojsgaard (2020), Ozias-Akins and Conner (2020), Scheben and Hojsgaard (2020), Schmidt (2020)). The only exception is represented by a very recent case concerning the transfer of the *PAR* allele from an apomictic dandelion to a sexual lettuce to induce egg cell division without fertilization (Underwood et al., 2022). Consequently, even in the modern era of functional genomics and systems biology, understanding the genetic control and molecular regulation of apomixis in plants appears much more complicated than expected.

This contribution addresses a critical review of the most cited articles published on apomixis in the pregenomic era (1980–2000) and genomic era (2001–2021). In genomics, the pregenomic and genomic eras refer to the time periods before and after the completion of the Human Genome Project. The working draft of the human genome was announced in 2000, and the papers describing it were published in February 2001 (Venter et al., 2001). Since then, the complete genomes of many reference organisms have been produced. These four decades are characterized by many scientific breakthroughs and methodological revolutions (e.g., availability of whole-genome and transcriptome assemblies, next-generation sequencing platforms, genome-editing tools and new breeding techniques). A massive set of cytological and ecological information, along with genetic and molecular data, has been collected mainly in model species (i.e., *Boechera holboellii*, *Hieracium* spp., *Hypericum perforatum*, *Paspalum* spp., *Ranunculus* spp., *Taraxacum officinale*), while Arabidopsis has often been used to elucidate the mechanisms of apomeiosis, parthenogenesis and apomixis. Several genes involved in the formation of unreduced embryo sacs and egg cells or responsible for the autonomous development of the embryo and endosperm have been cloned and characterized, but none of them were isolated in crop species (Pupilli and Barcaccia, 2012; Vijverberg et al., 2019; Hojsgaard, 2020; Ozias-Akins and Conner, 2020; Scheben and Hojsgaard, 2020; Schmidt, 2020). Hence, after four decades of substantial studies conducted in several laboratories with model plants, the asexual reproductive strategy called “gametophytic apomixis” still appears to be an unsolved puzzle. This situation has resulted in a loss of confidence by the major seed companies, making it difficult to acquire funds for conducting research on apomixis.

However, some novel views and original concepts are emerging. Noteworthy is the analogy between the Y-chromosome and apomixis-bearing chromosomes. Comparative genomic analyses have revealed common features such as the repression of recombination events, accumulation of transposable elements and degeneration of genes, from the most primitive to the most advanced evolutionary terms. Another emerging aspect is the link between apomixis and gene-specific silencing mechanisms, likely based on chromatin remodeling factors or transacting and heterochromatic interfering RNAs involved in both transcriptional and posttranscriptional gene regulation. More recently, merging lines of evidence regarding the role of auxin in cell fate specification of embryo sac and egg cell development have been reported in Arabidopsis, and some hypotheses have been postulated. From an evolutionary point of view, apomixis may now be regarded as a consequence of sexual failure rather than as a step toward clonal success (Albertini et al., 2019). Overall, these findings strengthen the hypothesis that apomixis as a whole may have evolved multiple times in angiosperm evolution following different developmental pathways deviating to different extents from sexuality (Barcaccia et al., 2020).

Here, the most cited publications on apomixis with the greatest potential impact on the scientific community working on this asexual mode of reproduction were analyzed. The goal was to make explicit their theoretical advances and/or practical applications and to depict the main trends in apomixis that have emerged from 20 years of studies in either the pregenomic or the genomic time period.

## Bibliometric Analysis

A bibliometric analysis of the studies related to apomixis was conducted in December 2021 based on Scopus database, and the search was limited to articles [LIMIT-TO (DOCTYPE, “ar”)] published between 1980 and 2021 in journals [LIMIT-TO (SRCTYPE, “j”)] written in the English language [LIMIT-TO (LANGUAGE, “English”)]. We first compared the results obtained by searching “apomixis” exclusively in the article title (TITLE) or considering the title, abstract, and keywords (TITLE-ABS-KEY). The former approach, more conservative, identified 280 articles (77 before 2000 and 203 after 2000). However, limiting the search for the term “apomixis” to the title alone led to the exclusion of several articles entirely related to asexual reproduction in plants. Conversely, extending the search of the term “apomixis” to the abstract and keywords increased the number of articles identified to 1,571, of which 377 were published before 2000 and 1,195 were published after 2000. However, some of these articles were not fully consistent with the topic. Considering that, from a practical point of view, it is challenging to manually double check the content of 1,571 articles, we analyzed the first 100 articles of each time frame (200 in total) to provide an estimated percentage of off-topic studies. A total of 12 and 11 articles from 1980–2000 and 2001–2021, respectively, were not related to apomixis in plants, as they mainly dealt with apomixis-like reproduction mechanisms in the animal kingdom. The geographical origin of these articles was random, and it is therefore quite unlikely that they could have an impact on the statistics presented in this study.

**FIGURE 1:**
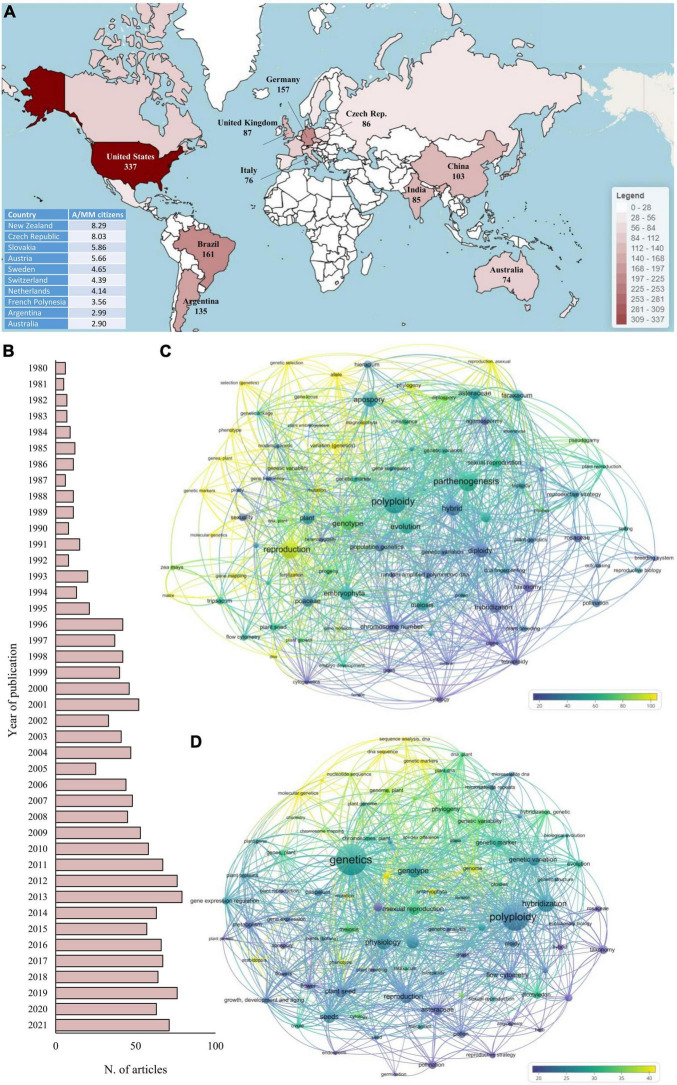
Bibliometric analysis of the studies related to apomixis retrieved from the Scopus database, where the search was limited to articles published in English-based journals between 1980 and 2021. The search of the term “apomixis” was limited to the title, abstract and keywords. **(A)** The 10 countries with the highest number of contributions (in terms of published articles) to apomixis. The bottom left table indicates the top 10 countries with the highest number of articles per million citizens A/MM. **(B)** Contributions to apomixis by year. **(C,D)** Co-occurrence network analysis of the 100 most used keywords in apomixis-related articles published in 1980–2000 and 2001–2021, respectively. The font size of each keyword is proportional to the number of times the keyword has been used within the articles considered, while the color scale is based on the average number of citations received by the documents in which each specific keyword occurs.

Based on these considerations, even if the second approach (i.e., searching “apomixis” within the title, abstract, or keywords) led to an overestimation of studies on apomixis, it was undoubtedly more comprehensive and was therefore employed for the selection of articles (Supplementary Tables 1, 2). Heatmapper was then used to visualize the geographical distribution of the contributors (Babicki et al., 2016; Figure 1A).

Historically, the United States, Brazil and Germany represent the countries with the highest number of contributions in the field of apomixis. They alone contributed 41.6% of all articles published in the last 40 years on this topic, with 655 articles out of 1,571. Even restricting the analysis to the last 10 years (2012–2021), these first three positions remain unchanged, confirming, in absolute terms, the consolidated leadership of these countries in this research area. Clearly, normalizing these data according to the number of researchers working in the natural sciences fields would provide a more precise idea of the efforts made by each country in this specific topic. However, retrieving this type of data is quite challenging, if not impossible. Instead, an attempt to normalize the data was made by considering the population of each country. In this case, the pattern was completely reversed, and countries such as New Zealand and the Czechia showed an article/citizen ratio four to ten times higher than that of the United States, Brazil and Germany.

In terms of time trends, the number of annual publications increased from 6 in 1980 to 75 in 2021, with a peak in 2013 (79), demonstrating a growing interest of researchers in this topic (Figure 1B). Notably, 22% (344) of all articles published in the last 40 years have been published in the last 5 years (2017–2021).

The keywords were also analyzed using a co-occurrence network analysis tool in VOSviewer software (van Eck and Waltman, 2010). To understand the dynamic change in their usage, the analysis was split into two sections considering the 100 most used keywords from 1980 to 2000 and from 2001 to 2021 (Figures 1C,D, respectively). The two keyword-based networks were organized considering both the use frequency of each keyword (as a function of the font size, namely, the larger the font size is, the greater the number of times the keyword has been used) and the average number of citations received by the documents in which each specific keyword occurred. Notably, excluding “apomixes,” the most used keyword in the last two decades was “genetics.” On the contrary, “genetics” never appeared in the ranking of the 100 most used keywords before 2000. However, in both periods, the articles containing keywords related to the field of genetics and molecular biology (e.g., “allele,” “genome,” “genetic markers,” and “DNA sequence”) were always the ones with the highest number of citations, highlighting the crucial role played by these two branches of biology in the study of apomixis. Apart from some laboratory analyses, i.e., cytometry, transversely used in the studies within the last 40 years, the use of keywords also reflected the evolution of the molecular methods used. From 1980 to 2000, the most popular keywords in the molecular genetics field were “allele,” “mutation” and “gene mapping,” while after 2000, studies were characterized by terms such as “genome,” “gene expression” and “microsatellite DNA.” The spread of capillary sequencers, NGS platforms and qPCR instruments at the turn of the century has shifted the focus of research toward more targeted and effective approaches to identifying the genetic determinants of apomixis.

Although not sufficiently exhaustive, the number of normalized citations per year (i.e., average number of citations/year) was used as an evaluation parameter in the choice and discussion of the most impactful articles on this topic. The absolute number of citations was not used, because it would have penalized the articles published in recent years. Finally, when choosing the most impactful articles (based on the average number of citations/year), we applied an additional filter based on consistency with the topic under consideration. As an example, the study by Silveira et al. (2009) is one of the articles with the highest average number of citations/year, but it does not provide information aimed at understanding apomictic mechanisms (i.e., it provides a list of reference genes to be used as control genes in qPCRs). For this reason, it was not included in the articles discussed in this review. Twenty articles were finally selected, 10 for each period.

## Pregenomic Era

Considering the 10 selected articles before 2000, we found that *Arabidopsis thaliana*, together with *Taraxacum*, represents the most studied plant species for scientific research on this topic. In the case of *Arabidopsis*, the scientific approaches used were commonly classical forward or reverse genetics with the analysis of mutants, gene mapping and functional characterization of candidate genes. Conversely, in *Taraxacum*, as in other species listed in the articles considered (i.e., *Erigeron annuus*, *Pennisetum squamulatum* or *Hieracium* spp.), more descriptive approaches were applied. In particular, microscopy, histology, flow cytometry, analysis of seed germination, plant genotyping by means of molecular markers (SSR, RAPD, AFLP or SCAR) have been frequently exploited. These tools were particularly useful to study the inheritance of apomixis, infer phylogenetic relationships between apomictic species or perform genetic mapping (Table 1).

**TABLE 1:** List of the 10 most cited publications on apomixis between 1980 and 2000 and between 2001 and 2021.

Taxon	Method/s	Main results	References	C/Y^1^
*Arabidopsis thaliana*	Mutagenesis, cytological analyses (electron and optical microscopy), and ploidy analysis (fluorescence microscopy)	Developmental and genetic characterization of *fis1* (fertilization-independent seed), *fis2*, and *fis3* mutants indicates that these genes normally have a controlling role in seed development after pollination and double fertilization.	Chaudhury et al., 1997	29.3
Angiosperm families (460)	Phylogenetic associations among reproductive-anomalous species using statistical tests	Apomictic, polysporic and polyembryonic species are polyploid or paleopolyploid and probably possess duplicate genes for female development. The authors also indicate that such species are associated at the familial level and are evolutionarily linked.	Carman, 1997	26.9
*Pennisetum squamulatum*	Molecular markers (RAPD and SCAR)	Genetic mapping of 13 molecular markers in an interspecific hybrid population of 397 individuals segregating for apomixis and sexuality. Surprisingly, 12 of the 13 markers strictly cosegregated with aposporous embryo sac development, clearly defining a contiguous apospory-specific genomic region in which no genetic recombination was detected.	Ozias-Akins et al., 1998	12.7
*Hieracium* spp.	Histological and cytological analyses (scanning electron microscopy, confocal microscopy)	Apomixis was observed in two distinct and nearly obligate *Hieracium* species and compared with seed formation in a sexual species. Novel features previously unrecorded in apomictic members of the *Hieracum* genus were described. The outcomes of apomictic events were further analyzed with respect to the types of seedlings that germinate from apomictic seeds and from hybrid seeds derived from crosses between the sexual and apomictic plants.	Koltunow et al., 1998	8.0
Melastomataceae family (11 species)	Pollen analysis	Controlled pollinations and observations of pollen tube growth, pollen fertility and cytological data were studied in 11 species of Melastomataceae. The apomictic species had lower pollen fertility than the sexual species, showing that low pollen fertility may be a useful indicator of apomixis if analyzed using careful sampling supplemented by emasculation experiments. The apomictic species also showed meiotic irregularities, probably related to hybridization, polyploidy and low pollen fertility.	Goldenberg and Shepherd, 1998	7.4
*Arabidopsis thaliana*	Gene mapping, mutant complementation	Using a map-based strategy, the authors cloned and sequenced the *F644* gene, showing that it encodes a SET-domain polycomb protein identical to MEDEA (MEA), which is encoded by a gene whose maternal-derived allele is required for embryogenesis.	Kiyosue et al., 1999	21.9
*Taraxacum* spp.	Flow cytometry; alloenzymes	The inheritance of apomixis was studied in narrow crosses between diploid sexuals and triploid apomicts from the *Taraxacum* section *Ruderalia*, and the reproductive mode of the offspring was investigated. Of the 26 allozyme-confirmed F1 hybrids, diploids were significantly less frequent than triploids. Seed set was not observed in diploids but was observed in one-third of the triploid hybrids and all of the tetraploid hybrids. Partial apomixis was caused by semisterility and not by residual sexuality (facultative apomixis).	Tas and Van Dijk, 1999	7.7
*Taraxacum* spp.	Cytological analyses; flow cytometry, SSR	Four non-apomictic diploid and 10 non-apomictic triploid hybrids were pollinated with diploids, and the progenies were analyzed. Seed fertility was significantly reduced in two diploid hybrids. The authors identified different types of progenies, concluded that elements of apomixis, diplospory and parthenogenesis can be uncoupled and suggested that several loci are involved in the genetic control of apomixis in *Taraxacum*.	Van Dijk et al., 1999	8.2
*Arabidopsis, Arabis, Hypericum* and *Poa* spp.	Flow cytometry	Seed samples of 32 species (obligate and facultative sexuals and apomicts of monocots and dicots) were investigated by flow cytometry to reveal the pathway of reproduction. The screen is suited to selecting sporophytic or gametophytic mutants in sexual species, identifying pure sexual or obligate apomictic genotypes from facultative apomictic species, and analyzing the inheritance of individual reproductive processes.	Matzk et al., 2000	22.7
*Erigeron annuus*	AFLPs	Diplospory and parthenogenesis are unlinked and inherited independently; the absence of agamospermy in diploid F1s appears to be best explained by a combination of recessive-lethal gametophytic selection against the parthenogenetic locus and univalent inheritance of the region bearing diplospory.	Noyes and Rieseberg, 2000	9.6
*Arabidopsis thaliana*	SSR markers; genetic engineering	Mutation of the *Arabidopsis* gene *DYAD*/*SWITCH1* (*SWI1*), a regulator of meiotic chromosome organization, leads to apomeiosis. The alteration of a single gene in a sexual plant can result in functional apomeiosis, a major component of apomixis.	Ravi et al., 2008	10.4
*Arabidopsis thaliana*	Cytology and flow cytometry; genetic markers	The creation of the MiMe genotype and apomeiosis phenotype: MiMe plants undergo mitotic-like division instead of normal meiotic division, without affecting subsequent sexual processes.	D’Erfurth et al., 2009	16.4
*Taraxacum officinale*	Chemical analysis; AFLP and MS-AFLP analysis	Stress-induced methylation changes are common and are mostly heritable. Sequence-independent, autonomous methylation variation is readily generated. This highlights the potential of epigenetic inheritance to play an independent role in evolutionary processes, which is superimposed on the system of genetic inheritance.	Verhoeven et al., 2010	33.7
*Arabidopsis thaliana*	Histological analysis; immunoblotting and immunoprecipitation; cloning and genomic analysis of small RNAs	The *Arabidopsis* protein ARGONAUTE 9 (AGO9) controls female gamete formation by restricting the specification of gametophyte precursors in a dose-dependent, non-cell-autonomous manner. *AGO9*-dependent sRNA silencing is crucial to specifying cell fate in the *Arabidopsis* ovule, and epigenetic reprogramming in companion cells is necessary for sRNA–dependent silencing in plant gametes.	Olmedo-Monfil et al., 2010	30.7
*Zea mays*	Genotyping; *in situ* mRNA hybridizations	The DNA methylation pathway active during reproduction is essential for gametophyte development in maize and plays a critical role in the differentiation between apomictic and sexual reproduction. Downregulation of an ovule-specific chromatin-based silencing pathway in maize would result in apomixis. Apomeiosis and parthenogenesis in *Tripsacum* are genetically linked.	Garcia-Aguilar et al., 2010	10.6
*Boechera* spp.	Cytohistological analyses; SuperSAGE analysis	Apomixis-specific gene expression is characterized by a significant overrepresentation of transcription factor activity. The link between hybridization and asexuality provides a hypothesis for multiple evolutionary origins of apomixis in the genus *Boechera*.	Sharbel et al., 2010	9.7
*Zea mays*	Cytology and immunochemistry; digital gene expression tag profiling	Female germ cell development in maize is dependent upon conserved small RNA pathways acting non-cell-autonomously in the ovule. Interfering with this repression leads to apomixis-like phenotypes in maize. AGO104 influences the transcription of many targets in the ovaries, with a strong effect on centromeric repeats.	Singh et al., 2011	14.9
*Pennisetum squamulatum*	Flow cytometry; genetic engineering; histological analysis	The *PsASGR-BABY BOOM*-like (*PsASGR-BBML*) gene is expressed in egg cells before fertilization and can induce parthenogenesis and the production of haploid offspring in transgenic sexual pearl millet. A reduction in *PsASGR-BBML* expression in apomictic F1 transgenic plants results in fewer visible parthenogenetic embryos and a reduction in the embryo cell number compared with controls.	Conner et al., 2015	12.0
*Taraxacum officinale*	Flow cytometry; SSR, AFLP; demethylation experiment	Variations in DNA methylation contribute to heritable differences in flowering time within a single widespread apomictic clonal lineage of the common dandelion. Epigenetic mechanisms can facilitate adaptive divergence within genetically uniform asexual lineages.	Wilschut et al., 2016	8.5
*Citrus* spp.	DNA sequencing; bulk segregant analysis (BSA); local gene-based association analysis for the polyembryony locus; transcriptome sequencing	A comparative population analysis suggested that genomic regions harboring energy- and reproduction-associated genes are probably under selection in cultivated citrus. The genetic locus responsible for citrus polyembryony, a form of apomixis, is an 80-kb region containing 11 candidate genes; one of them, *CitRWP*, is expressed at higher levels in ovules of polyembryonic cultivars. A miniature inverted-repeat transposable element insertion in the promoter region of *CitRWP* cosegregated with polyembryony.	Wang et al., 2017	32.2

*^1^C/Y, citation/year.*

*For each publication, the taxon used, the main methods involved, the main findings, the reference and the average number of citations per year are reported.*

Concerning basic research on the possible molecular mechanisms underlying apomixis, it is worth mentioning the efforts of Kiyosue et al. (1999). They isolated a mutation in *Arabidopsis*, *f644*, that allows for replication of the central cell and subsequent endosperm development without fertilization. By using a map-based strategy, the authors cloned and sequenced the *F644* gene and showed that it encodes a SET-domain polycomb protein. The latter, in turn, is the identical protein codified by *MEDEA* (*MEA*), a pivotal gene whose maternally derived allele is required for embryogenesis. These findings demonstrated the functions for plant polycomb proteins in the suppression of central cell proliferation and endosperm development. In another article, Chaudhury et al. (1997) analyzed other Arabidopsis mutants, *fis1*, *fis2* and *fis3*, in which certain processes of seed development were uncoupled from the double-fertilization event that occurs after pollination. The authors proposed a model in which the FIS1-FIS2-FIS3 interaction blocks endosperm development by acting in different stages of seed development. Their proposal assumed that this process in wild-type plants is initiated by a pollination-induced inactivation of the FIS1–FIS2–FIS3 complex (Chaudhury et al., 1997). The high number of citations per year reflects the importance of these two articles.

Another highly cited article, representing a milestone in apomixis research, is the contribution by Carman in 1997. He analyzed around 460 families of angiosperms and validated the hypothesis that the partial-to-complete replacement of meiosis by embryo sac formation in apomictic and polysporic species results from asynchronously expressed duplicated genes that control female development (Carman, 1997). Also, Carman further suggested that asexual reproduction in apomicts preserves primary genomes whilst sexual reproduction in polysporic and polyembryonic polyploids accelerates paleopolyploidization. Paleopolyploidization may sometimes eliminate the gene duplications required for apomixis while retaining those duplications required for polyspory or polyembryony.

Among the various scientific contributions listed in Table 1 are the studies that have provided screening tools to determine the reproductive pathways in monocotyledons and dicotyledons. An emblematic example is the work of Matzk et al. The authors analyzed seed samples collected from 32 monocot and dicot species (apomicts along with obligate and facultative sexuals) *via* flow cytometry. Based on the cytometry estimates, they proposed a novel screen for the route of reproduction based on the ploidy levels of embryo and endosperm nuclei from mature seeds (Matzk et al., 2000).

The use of molecular markers for mapping gene loci involved in apomictic phenomena represents one of the most used approaches in the pregenomic era. For example, Ozias-Akins et al. by analyzing an interspecific hybrid population of 397 individuals that were segregated for apomixis and sexuality, identified 13 predictive markers (Ozias-Akins et al., 1998). Twelve of the 13 markers strictly cosegregated with aposporous embryo sac development, clearly defining a contiguous apospory-specific genomic region in which no genetic recombination was detected. Noyes and Rieseberg investigated the genetic basis of agamospermy in a segregating population of 130 F1 hybrids obtained from a cross between triploid agamospermous *Erigeron annuus* (2n = 27) and sexual diploid *E. strigosus* (2n = 18) (Noyes and Rieseberg, 2000). By means of linkage analyses performed on 387 segregating amplified fragment length polymorphisms (AFLPs), they found that four closely linked markers with polysomic inheritance were significantly associated with parthenogenesis. Moreover, they identified 11 co-segregating markers with univalent inheritance completely associated with diplospory.

Cytological analyses based on microscopy represent another essential tool in the study of apomixis. This is the case for the contribution by Koltunow in *Hieracium* and that of Goldenberg and Shepherd in the *Melastomataceae* family. In the first case, Koltunow et al. performed a detailed cytological study of two distinct and nearly obligate species, *H. aurantiacum* and *H. piloselloides*. Also, they compared apomixis with seed formation in a sexual species to define the cellular basis for apomixis. In the light of this, they described novel features previously unrecorded in apomictic members of this genus and provided the basis for further molecular, cell biological and mechanistic studies investigating the control of apomixis (Koltunow et al., 1998). In the second study, controlled pollinations and observations of pollen tube growth, pollen fertility and cytological data were studied in 11 species of Melastomataceae (Goldenberg and Shepherd, 1998). The apomictic species had lower pollen fertility than the sexual ones, showing that low pollen fertility may be a useful indicator of apomixis if analyzed using careful sampling supplemented by emasculation experiments. The apomictic species also showed meiotic irregularities that were probably related to hybridization, polyploidy and low pollen fertility.

In *Taraxacum*, the inheritance of apomixis was studied in narrow crosses between diploid sexuals and triploid apomicts from the *Taraxacum* section *Ruderalia*, and the reproductive mode of the offspring was investigated. Of the 26 allozyme-confirmed F1 hybrids, diploids were significantly less frequent than triploids (Tas and Van Dijk, 1999). Seed set was not observed in any of the diploids, but it was observed in one-third of the triploid hybrids and in all the tetraploid hybrids. In a second article, the same authors crossed diploid sexual plants with four non-apomictic diploid and 10 non-apomictic triploid hybrids (obtained in a previous study) and analyzed the resulting progenies. The authors identified different types of progenies and concluded that elements of apomixis, diplospory and parthenogenesis can be uncoupled. This result is inconsistent with the single-locus model for apomixis in *Taraxacum* and suggests that several loci are involved in the genetic control of apomixis in *Taraxacum* (Van Dijk et al., 1999).

## Genomic Era

*Pennisetum*, *Taraxacum* and especially *Arabidopsis* continue to play a major role in post-2000 studies. In particular, *Arabidopsis* has been at the center of tremendous advances in our understanding of several fields, providing valuable information regarding the role of some candidate genes controlling apomixis. However, it should also be noted that crop species, such as corn and citrus, are starting to be studied. The identification of key genes involved in apomictic mechanisms in polyploids or interspecific hybrids has only been possible due to the introduction of genetic engineering techniques and sequencing platforms. Moreover, differently from the pregenomic era, the most cited articles in the post-2000 era almost exclusively addressed the functional characterization of genes and the *ad hoc* constitution of mutants. Finally, new hypotheses were proposed on the role of epigenetics, DNA methylation, miRNAs or siRNAs in reproductive mechanisms.

One of the milestones in the genetic study of apomixis is represented by the identification of the *DYAD/SWITCH1* (*SWI1*) locus, a regulator of meiotic chromosome organization, whose *dyad* allele leads to unreduced female gamete formation (apomeiosis). Most of the fertile diploid ovules were successfully fertilized by haploid pollen producing triploid seeds in dyad plants (Ravi et al., 2008). Another *Arabidopsis* mutant producing functional diploid gametes genetically identical to their mother was generated by combining three different mutations that worked to fully replace meiosis with a mitotic-like division. In particular, the mutated alleles characterizing the *MiMe* mutant (i.e., *MI*tosis instead of *ME*iosis) were *osd1*, *Atspo11-1* and *Atrec8*. The latter two led to a mitotic-like first meiotic division (by affecting the homologous recombination and the orientation of sister chromatids), while *osd1* prevented the second meiotic division. In contrast to the *dyad* mutation, both male and female gametes were unreduced such that selfed *MiMe* progeny were commonly tetraploid, while backcrossing diploid *MiMe* and wild-type plants produced triploid plants regardless of the use of male or female *MiMe* gametes (D’Erfurth et al., 2009). Further upstream, *AGO9*, a member of the ARGONAUTE family that is known to interact with miRNAs and siRNAs, was found to regulate early cell specification in the ovule. In particular, mutations in *AGO9* lead to the production of multiple abnormal somatic cells, some of which are able to initiate gametogenesis without undergoing meiosis (Olmedo-Monfil et al., 2010). When a single cell undergoes meiosis, it gives rise to a functional haploid megaspore. Also, identical results were obtained by mutating *SUPPRESSOR OF GENE SILENCING 3* (*SGS3*) and *RNA-DEPENDENT RNA POLYMERASE 6* (*RDR6*). The latter converts the ssRNA precursors of *trans-*acting siRNAs (ta-siRNAs) into dsRNA, while *SGS3* encodes an RNA-binding protein involved in the same pathway.

Strong homology of the components involved in silencing *via* DNA methylation was established between *Arabidopsis* and maize. Six loci that are specifically downregulated in ovules of apomictic plants were identified in maize-*Tripsacum* hybrids. Four of them encode chromatin-modifying enzymes (CMEs) that have been predicted (CHR106, DMT103, and DMT105) or shown (DMT102) to regulate DNA methylation. More precisely, CHR106 participates in the maintenance of methylation at both CG and non-CG sites, while the DNA methyltransferases DMT102, DMT105 and DMT103 act redundantly to maintain non-CG methylation. The set of deregulated genes also included *hdt104*, a member of the plant-specific histone deacetylase family, and *hon101*, a histone H1 linker protein gene. Specifically, loss-of-function alleles for *dmt102* and *dmt103*, homologous to the *Arabidopsis* CHROMOMETHYLASE and DOMAINS REARRANGED METHYLTRANSFERASE families, revealed apomictic phenotypes with unreduced gametes and the formation of multiple embryo sacs in the ovule (Garcia-Aguilar et al., 2010). Subsequent homology with *Arabidopsis* was identified as a dominant mutation resulting in the formation of functionally unreduced gametes in maize. *AGO104* is related to *Arabidopsis AGO9*; however, while *AGO9* acts to repress germ cell fate in somatic tissues, *AGO104* acts to repress somatic fate in germ cells. *AGO104* accumulates specifically in somatic cells surrounding the female meiocyte, suggesting a mobile signal rather than cell-autonomous control. Furthermore, *AGO104* is necessary for non-CG methylation of centromeric and knob-repeat DNA and influences the transcription of many targets in the ovaries, with a strong effect on centromeric repeats (Singh et al., 2011). Another perspective affecting the onset of DNA methylation was reported for dandelions. Environmental stresses can trigger methylation changes, which may have evolutionary consequences, even in the absence of sequence variation. These changes are evidence that specific stresses can trigger specific methylation changes and that most of the induced changes are faithfully transmitted to offspring (Verhoeven et al., 2010). Further studies on dandelions substantiated the role of the methylation polymorphism during functional flowering time, confirming that epigenetic variation contributes to heritable phenotypic divergence in ecologically relevant traits in natural plant populations (Wilschut et al., 2016). Therefore, epigenetic variation plays a key role in the adaptive potential of populations to rapidly face changing environments or when genetic variation is limited. Additionally, a transcriptomic analysis supported the hypothesis of multiple evolutionary origins of apomixis and a possible link with hybridization was noted in an ancient hybrid of *Boechera* exhibiting a diplosporous type of *apomixis (Sharbel et al., 2010*). Apomixis-specific gene expression was characterized by a significant overrepresentation of transcription factor activity, showing that apomeiosis is associated with downregulation at the megaspore mother cell stage. On the other hand, apospory in *Pennisetum squamulatum*, a member of the Poaceae family, segregates *via* a single dominant locus transmitted by a large, hemizygous, non-recombining chromosomal region, the apospory-specific genomic region (*ASGR*). The ASGR contains multiple copies of the *PsASGR-BABY BOOM-like* (*PsASGR-BBML*) gene, a member of the BBM-like subgroup of the *APETALA 2/ETHYLENE RESPONSE FACTOR* (*AP2/ERF*) DNA-binding domain family. Additionally, the function of the *PsASGR-BBML* transgene was discovered: this gene is expressed in egg cells before fertilization and induces both parthenogenesis and the production of haploid offspring in sexual pearl millet (Conner et al., 2015). While the studies reported thus far have focused on gametophytic apomixis, asexual reproduction in the *Citrus* genus is largely based on sporophytic apomixis. Accessions of primitive, wild relative and cultivated *Citrus* species have been sequenced to shed light on the genetic locus responsible for polyembryony, resulting in 11 candidate genes. One of them, *CitRWP*, is expressed at higher levels in ovules of polyembryonic cultivars (Wang et al., 2017). *CitRWP*, also denoted *Cg4g018970*, encodes an RWP-RK domain similar to the *Arabidopsis* RKD family of proteins and acts as a regulator of egg cell-related genes. The extensive transcriptomic analysis by Wang et al. led to important insights suggesting that nucellar embryogenesis and sexual reproduction may employ similar genes or pathways.

## Conclusion

Apomixis has aroused great scientific and intellectual curiosity, leading to an exponential increase in research studies. The prospect of introducing apomixis “as a whole” in crop species still remains the main goal, but the attempt to engineer species of agronomic interest has been unsuccessful. In terms of model species, the scenario has remained broadly unchanged since the 1980s: most studies are conducted on a few species, although in recent years, there have been some attempts to extend this research to agronomically important crops. In terms of methodologies, few clear differences were found between the pregenomic and genomic eras. The use of genetic engineering and sequencing platforms has begun to spread in the study of apomixis, but some methodologies, including flow cytometry and microscopy, still represent important research activities. Identification of the master regulatory switch triggering apomixis in sexual crops is a goal for plant breeders. The massive number of studies conducted in apomixis in the last 40 years requires a critical analysis aimed at integrating all the information produced. A new scenario is taking shape, and the exponential accumulation of knowledge related to apomictic mechanisms in model species is finally reaching application potential: genome editing. It is still too early to predict whether this new technology represents the beginning of a new era, but it would not be surprising to see that the 10 most cited articles in the next 20 years mainly concern the use of genomic editing approaches to transfer what has been learned thus far in model species into crops.

## Author Contributions

All authors listed have made a substantial, direct, and intellectual contribution to the work, and approved it for publication.

## Conflict of Interest

The authors declare that the research was conducted in the absence of any commercial or financial relationships that could be construed as a potential conflict of interest.

## Publisher’s Note

All claims expressed in this article are solely those of the authors and do not necessarily represent those of their affiliated organizations, or those of the publisher, the editors and the reviewers. Any product that may be evaluated in this article, or claim that may be made by its manufacturer, is not guaranteed or endorsed by the publisher.
